# Supplementary material for: Toxoplasma gondii chronic infection decreases visceral nociception through peripheral opioid receptor signaling
Source: PLoS Pathog. 2025 Apr 29;21(4):e1013106. doi: 10.1371/journal.ppat.1013106 (PMC12068698; doi:10.1371/journal.ppat.1013106)
Supplement: S1 Fig — (A and B) Unsupervised analysis with flow cytometry data obtained from the colon of non-infected or ip-infected mice at acute stage (14 days post-infection) was performed using OMIQ software. Statistical analysis was performed using a Mann-Whitney test on each subpopulation and summarized into one single graph. (A) Unsupervised analysis was performed on live CD45+ cells negative for CD3ε, CD19 and EpCAM (called Lin-negative cells). Opt-SNE plot of 34 000 Lin-negative cells concatenated from 4 non-infected mice (left) and 42 500 Lin-negative cells concatenated from 5 infected mice (right), divided in 9 clusters using Phenograph algorithm, based on the expression of surface markers associated with each cell subset. Table under opt-sne shows the relative abundance of each cluster in each condition (infected (orange) or not (white)). (B) Unsupervised analysis was performed on live T cells using the following gating strategy: EpCAM-/ CD45+/ CD3+. Opt-SNE plot of 36 360 colonic T cells concatenated from 4 non-infected mice (left) and 45 450 colonic T cells concatenated from 5 infected mice (right), divided in 15 clusters using Phenograph algorithm, based on the expression of different markers. Table under opt-sne shows the relative abundance of each cluster in each condition (infected (orange) or not (white)). (PDF) [file ppat.1013106.s001.pdf]

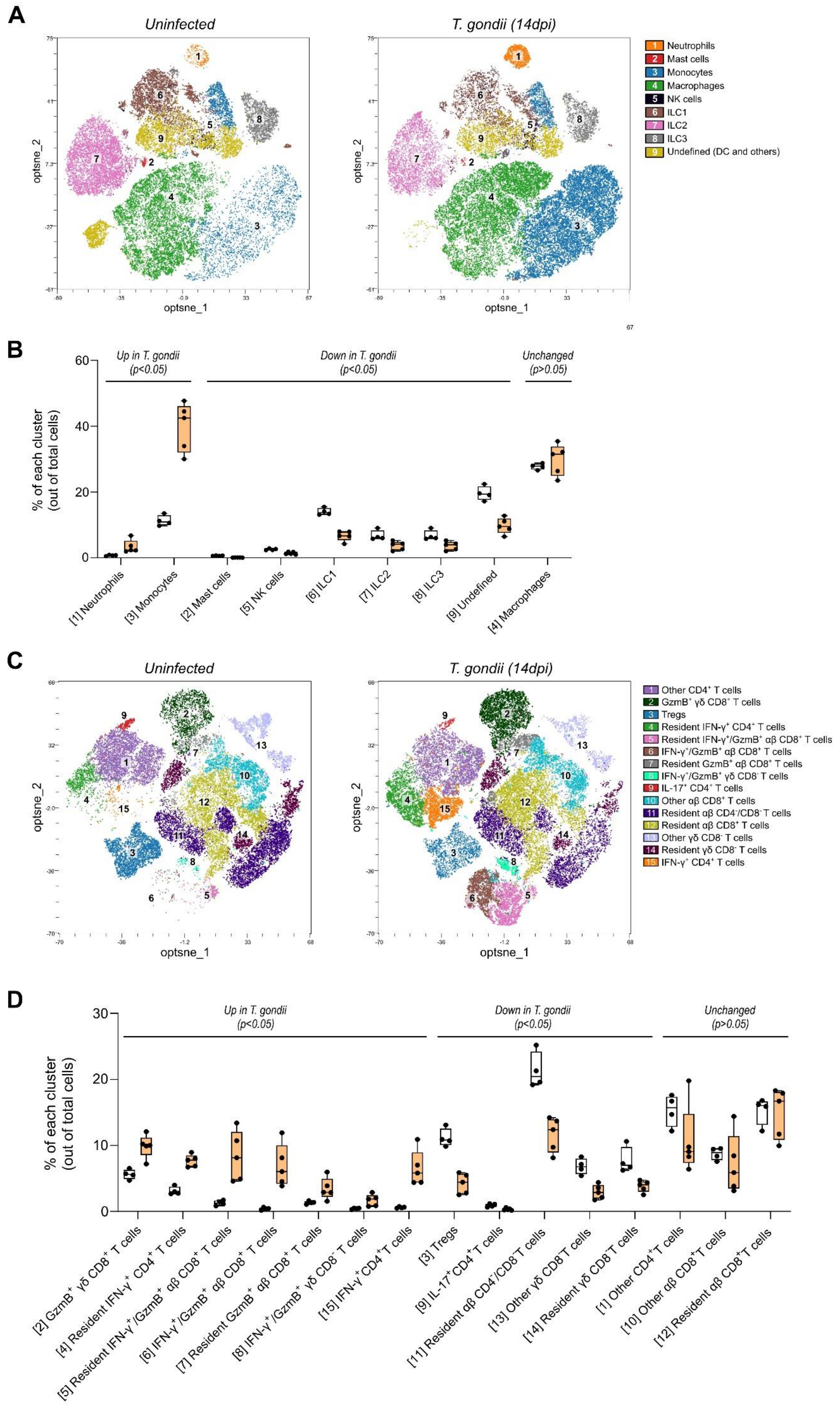
A
Uninfected
T. gondii (14dpi)
1 Neutrophils
2 Mast cells
3 Monocytes
4 Macrophages
5 NK cells
6 ILC1
7 ILC2
8 ILC3
9 Undefined (DC and others)
optsne_1
optsne_2
B
Up in T. gondii
(p<0.05)
Down in T. gondii
(p<0.05)
Unchanged
(p>0.05)
% of each cluster
(out of total cells)
[1] Neutrophils
[3] Monocytes
[2] Mast cells
[5] NK cells
[6] ILC1
[7] ILC2
[8] ILC3
[9] Undefined
[4] Macrophages
C
Uninfected
T. gondii (14dpi)
1 Other CD4⁺ T cells
2 GzmB⁺ γδ CD8⁺ T cells
3 Tregs
4 Resident IFN-γ⁺ CD4⁺ T cells
5 Resident IFN-γ⁺/GzmB⁺ αβ CD8⁺ T cells
6 IFN-γ⁺/GzmB⁺ αβ CD8⁺ T cells
7 Resident GzmB⁺ αβ CD8⁺ T cells
8 IFN-γ⁺/GzmB⁺ γδ CD8⁻ T cells
9 IL-17⁺ CD4⁺ T cells
10 Other αβ CD8⁺ T cells
11 Resident αβ CD4⁻/CD8⁻ T cells
12 Resident αβ CD8⁺ T cells
13 Other γδ CD8⁻ T cells
14 Resident γδ CD8⁻ T cells
15 IFN-γ⁺ CD4⁺ T cells
optsne_1
optsne_2
D
Up in T. gondii
(p<0.05)
Down in T. gondii
(p<0.05)
Unchanged
(p>0.05)
% of each cluster
(out of total cells)
[2] GzmB⁺ γδ CD8⁺ T cells
[4] Resident IFN-γ⁺ CD4⁺ T cells
[5] Resident IFN-γ⁺/GzmB⁺ αβ CD8⁺ T cells
[6] IFN-γ⁺/GzmB⁺ αβ CD8⁺ T cells
[7] Resident GzmB⁺ αβ CD8⁺ T cells
[8] IFN-γ⁺/GzmB⁺ γδ CD8⁻ T cells
[15] IFN-γ⁺ CD4⁺ T cells
[3] Tregs
[9] IL-17⁺ CD4⁺ T cells
[11] Resident αβ CD4⁻/CD8⁻ T cells
[13] Other γδ CD8⁻ T cells
[14] Resident γδ CD8⁻ T cells
[1] Other CD4⁺ T cells
[10] Other αβ CD8⁺ T cells
[12] Resident αβ CD8⁺ T cells

S1 Fig. Acute *T. gondii* infection induces recruitment of myeloid cells and T cells in the colon

(**A and B**) Unsupervised analysis with flow cytometry data obtained from the colon of non-infected or ip-infected mice at acute stage (14 days post-infection) was performed using OMIQ software. Statistical analysis was performed using a Mann-Whitney test on each subpopulation and summarized into one single graph. (**A**) Unsupervised analysis was performed on live $CD45^+$ cells negative for CD3ε, CD19 and EpCAM (called Lin-negative cells). Opt-SNE plot of 34 000 Lin-negative cells concatenated from 4 non-infected mice (left) and 42 500 Lin-negative cells concatenated from 5 infected mice (right), divided in 9 clusters using Phenograph algorithm, based on the expression of surface markers associated with each cell subset. Table under opt-sne shows the relative abundance of each cluster in each condition (infected (orange) or not (white)). (**B**) Unsupervised analysis was performed on live T cells using the following gating strategy: $EpCAM^-$ / $CD45^+$ / $CD3^+$. Opt-SNE plot of 36 360 colonic T cells concatenated from 4 non-infected mice (left) and 45 450 colonic T cells concatenated from 5 infected mice (right), divided in 15 clusters using Phenograph algorithm, based on the expression of different markers. Table under opt-sne shows the relative abundance of each cluster in each condition (infected (orange) or not (white)).
