# Supplementary material for: Toxoplasma gondii chronic infection decreases visceral nociception through peripheral opioid receptor signaling
Source: PLoS Pathog. 2025 Apr 29;21(4):e1013106. doi: 10.1371/journal.ppat.1013106 (PMC12068698; doi:10.1371/journal.ppat.1013106)
Supplement: S2 Fig — (A and B) Cell doublets were excluded using double gating on SSC-A vs. SSC-H followed by SSC-W vs. SSC-H exclusion. Dead cells were removed using a viability marker to allow analysis of live cells only. Arrows indicate the order of the gating strategy. (A) Gating strategy used to identify the different T cell subsets in the colon. (B) Gating strategy used to identify the different cell subsets belonging to the myeloid and Innate Lymphoid Cells (ILC) lineages. (PDF) [file ppat.1013106.s002.pdf]

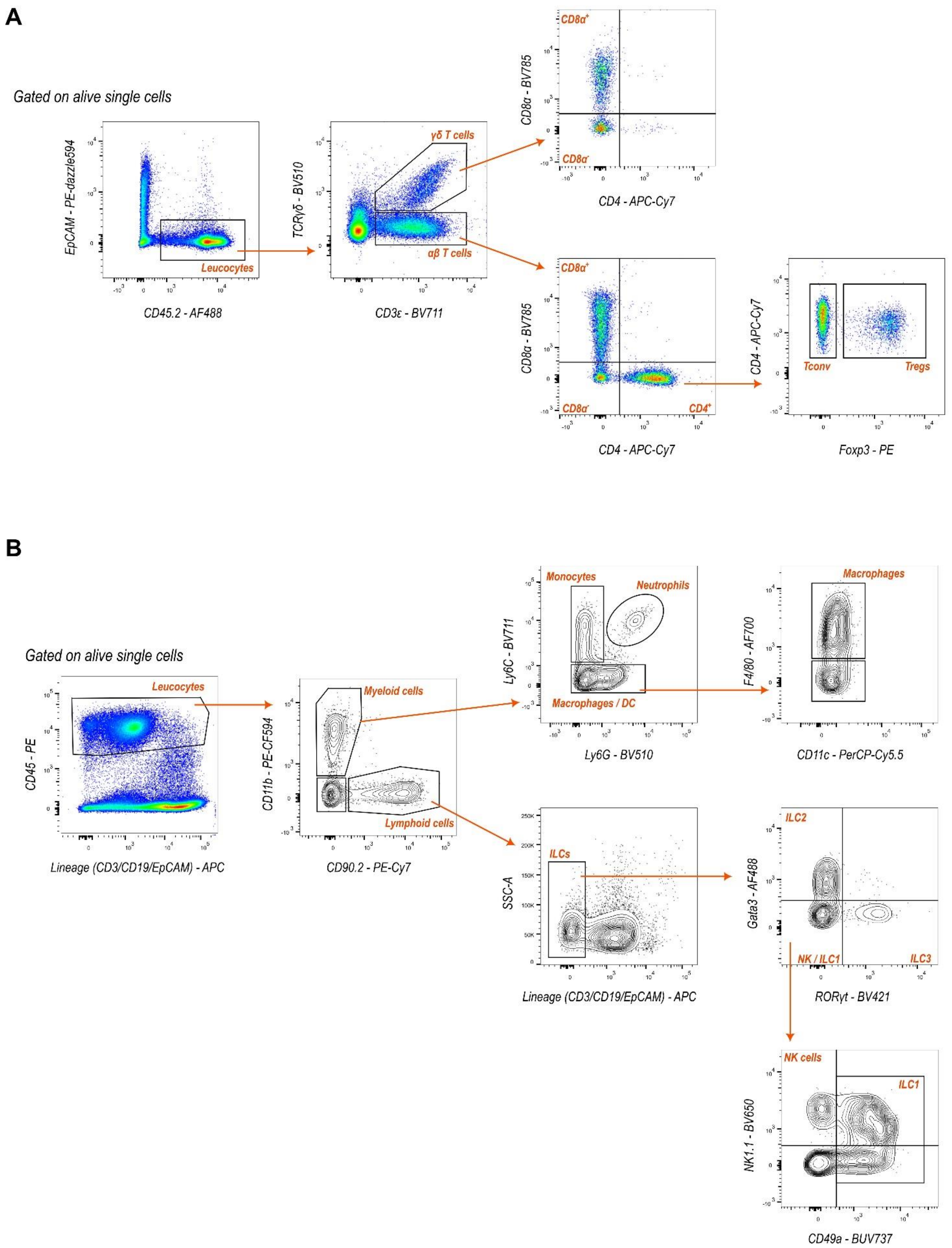

S2 Fig (related to Fig 2). Gating strategies used to identify the immune populations in the colon

(**A and B**) Cell doublets were excluded using double gating on SSC-A vs. SSC-H followed by SSC-W vs. SSC-H exclusion. Dead cells were removed using a viability marker to allow analysis of live cells only. Arrows indicate the order of the gating strategy. (**A**) Gating strategy used to identify the different T cell subsets in the colon. (**B**) Gating strategy used to identify the different cell subsets belonging to the myeloid and Innate Lymphoid Cells (ILC) lineages.
