# Supplementary material for: Toxoplasma gondii chronic infection decreases visceral nociception through peripheral opioid receptor signaling
Source: PLoS Pathog. 2025 Apr 29;21(4):e1013106. doi: 10.1371/journal.ppat.1013106 (PMC12068698; doi:10.1371/journal.ppat.1013106)
Supplement: S3 Fig — (A) Expression of the 3 opioid encoding - genes (i.e., Pomc, Penk and Pdyn) was measured by RT-qPCR in the whole colon of mice chronically ip-infected for 70 days. Values for gene expression were calculated based on the difference between the housekeeping gene Hprt and the gene of interest (ΔCt). Data correspond to a pool of 2 independent experiments and each dot represents one mouse. (B) Mouse model for T cell-specific deletion of enkephalin-encoding gene (Penk) in mice. Penk-floxed mice carrying LoxP sites upstream and downstream of Penk exon 2 were crossed with transgenic mice expressing the Cre recombinase under the control of CD4 promoter (CD4-Cre). Cartoon was modified from https://openclipart.org/detail/17558/simple-cartoon-mouse. (C) Deletion of Penk was confirmed by measuring the expression of Penk mRNA by flow cytometry (FlowFISH) in naïve (CD62L + /CD44-) or activated (CD44+) conventional (FoxP3-) CD4 + T cells, and in regulatory CD4 + T cells (Foxp3+). (D) Experimental workflow: C57BL/6 expressing (Penk TWT) or not (Penk TKO) in T cells were infected with the Pru.GFP.GRA6-OVA T. gondii by intraperitoneal injection with 200 tachyzoïtes. At 10 weeks post-infection, colorectal distension was performed to assess visceral sensitivity. Cartoon was modified from https://openclipart.org/detail/17558/simple-cartoon-mouse. (E) Visceromotor response (VMR) to increasing colorectal distension pressure (15–60 mm Hg) was measured in chronically ip-infected mice invalidated (Penk TKO) or not (Penk TWT) for Penk gene in T cells. VMR are represented with mean + /- SEM with n = 7 Penk TWT vs 6 Penk TKO animals. Statistical analysis was performed on Areas Under the Curve (AUC) with a Mann-Whitney test using GraphPad Prism. Data correspond to one experiment. (F) Parasite loads in the brain were measured by qPCR on genomic DNA for each mouse, showing no difference between Penk TWT and Penk TKO infected mice. (PDF) [file ppat.1013106.s003.pdf]

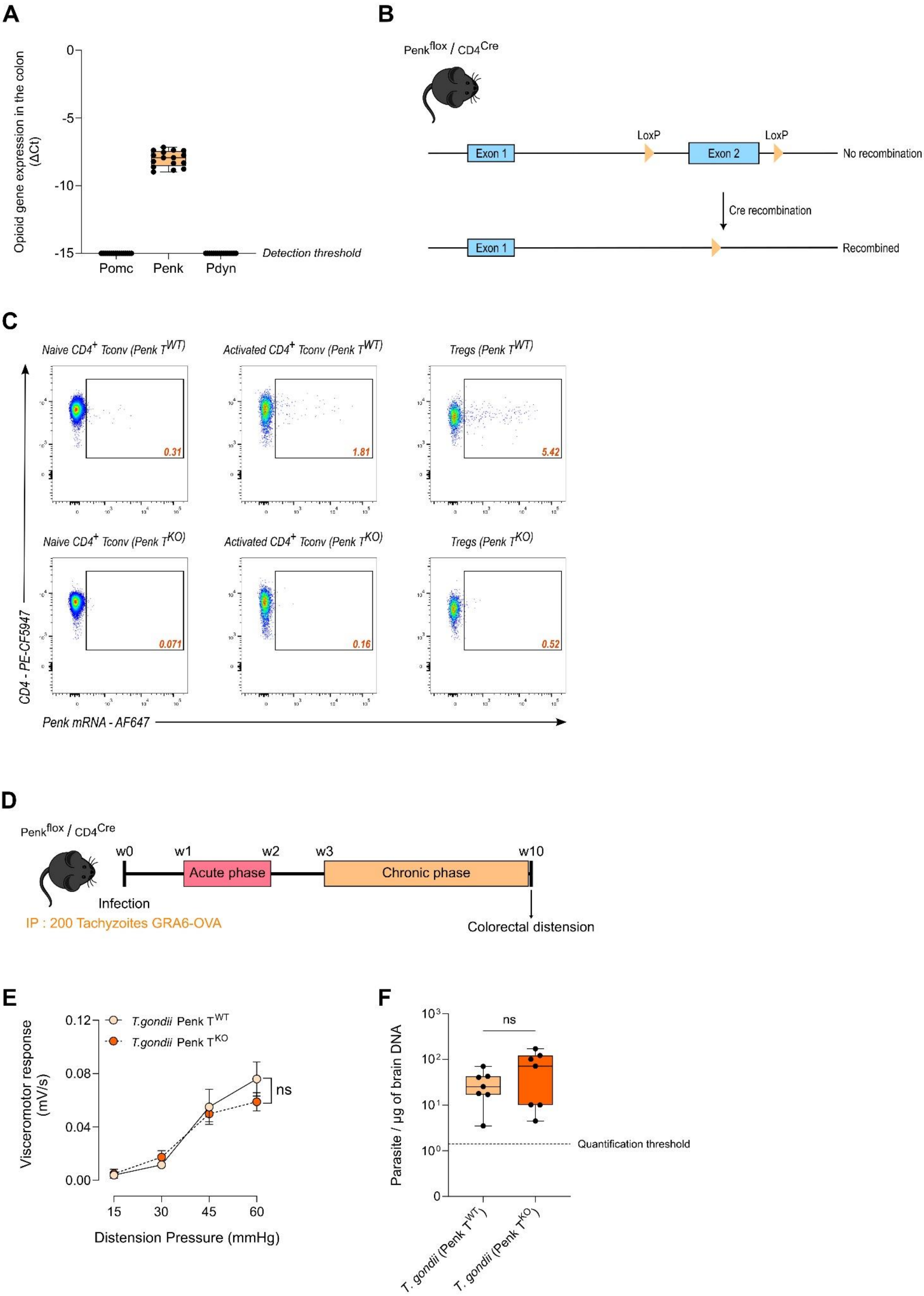
A
Opioid gene expression in the colon (ΔCt)
0
-5
-10
-15
Pomc
Penk
Pdyn
Detection threshold
B
Penk flox / CD4 Cre
Exon 1
LoxP
Exon 2
LoxP
No recombination
Cre recombination
Exon 1
Recombined
C
Naive CD4+ Tconv (Penk T WT)
Activated CD4+ Tconv (Penk T WT)
Tregs (Penk T WT)
0.31
1.81
5.42
Naive CD4+ Tconv (Penk T KO)
Activated CD4+ Tconv (Penk T KO)
Tregs (Penk T KO)
0.071
0.16
0.52
CD4 - PE-CF5947
Penk mRNA - AF647
D
Penk flox / CD4 Cre
w0
w1
w2
w3
w10
Acute phase
Chronic phase
Infection
IP : 200 Tachyzoites GRA6-OVA
Colorectal distension
E
Visceromotor response (mV/s)
0.12
0.08
0.04
0.00
T.gondii Penk T WT
T.gondii Penk T KO
ns
15
30
45
60
Distension Pressure (mmHg)
F
Parasite / µg of brain DNA
10³
10²
10¹
10⁰
0
ns
Quantification threshold
T. gondii (Penk T WT)
T. gondii (Penk T KO)

S3 Fig (related to Fig 4): *T. gondii*-induced decrease in nociceptive responses is independent of T-cell derived enkephalins

(A) Expression of the 3 opioid encoding - genes (i.e. *Pomc, Penk* and *Pdyn*) was measured by RT-qPCR in the whole colon of mice chronically ip-infected for 70 days. Values for gene expression were calculated based on the difference between the housekeeping gene *Hprt* and the gene of interest (ΔCt). Data correspond to a pool of 2 independent experiments and each dot represents one mouse. **(B)** Mouse model for T cell-specific deletion of enkephalin-encoding gene (*Penk*) in mice. *Penk*-floxed mice carrying LoxP sites upstream and downstream of *Penk* exon 2 were crossed with transgenic mice expressing the Cre recombinase under the control of CD4 promoter (CD4-Cre). Cartoon was modified from https://openclipart.org/detail/17558/simple-cartoon-mouse. **(C)** Deletion of Penk was confirmed by measuring the expression of Penk mRNA by flow cytometry (FlowFISH) in naïve (CD62L+/CD44-) or activated (CD44+) conventional (FoxP3-) CD4+ T cells, and in regulatory CD4+ T cells (Foxp3+). **(D)** Experimental workflow: C57BL/6 expressing (Penk T^{WT}) or not (Penk T^{KO}) in T cells were infected with the Pru.GFP.GRA6-OVA *T. gondii* by intraperitoneal injection with 200 tachyzoïtes. At 10 weeks post-infection, colorectal distension was performed to assess visceral sensitivity. Cartoon was modified from https://openclipart.org/detail/17558/simple-cartoon-mouse. **(E)** Visceromotor response (VMR) to increasing colorectal distension pressure (15 to 60 mm Hg) was measured in chronically ip-infected mice invalidated (Penk T^{KO}) or not (Penk T^{WT}) for Penk gene in T cells. VMR are represented with mean +/- SEM with n = 7 Penk T^{WT} vs 6 Penk T^{KO} animals. Statistical analysis was performed on Areas Under the Curve (AUC) with a Mann-Whitney test using GraphPad Prism. Data correspond to one experiment. **(F)** Parasite loads in the brain were measured by qPCR on genomic DNA for each mouse, showing no difference between Penk T^{WT} and Penk T^{KO} infected mice.
