# Supplementary material for: Toxoplasma gondii chronic infection decreases visceral nociception through peripheral opioid receptor signaling
Source: PLoS Pathog. 2025 Apr 29;21(4):e1013106. doi: 10.1371/journal.ppat.1013106 (PMC12068698; doi:10.1371/journal.ppat.1013106)
Supplement: S4 Fig — (A and B) Colorectal distension was performed to assess visceral sensitivity of each mouse before (in white) or 30 min after (in gray) intraperitoneal injection of naloxone-methiodide. (A) Visceromotor response (VMR) in response to increasing colorectal distension pressure (15–60mmHg) was measured in uninfected mice (ni) treated or not with Naloxone methiodide. Data are shown as mean + /- SEM and are from 1 experiment representative with n = 6 mice. (B) Area Under the Curve (AUC) are represented for each mouse with 2 dots corresponding to the AUC before (in white) and after (in gray) naloxone methiodide treatment. Statistical analysis was performed using a Wilcoxon’s test. (PDF) [file ppat.1013106.s004.pdf]

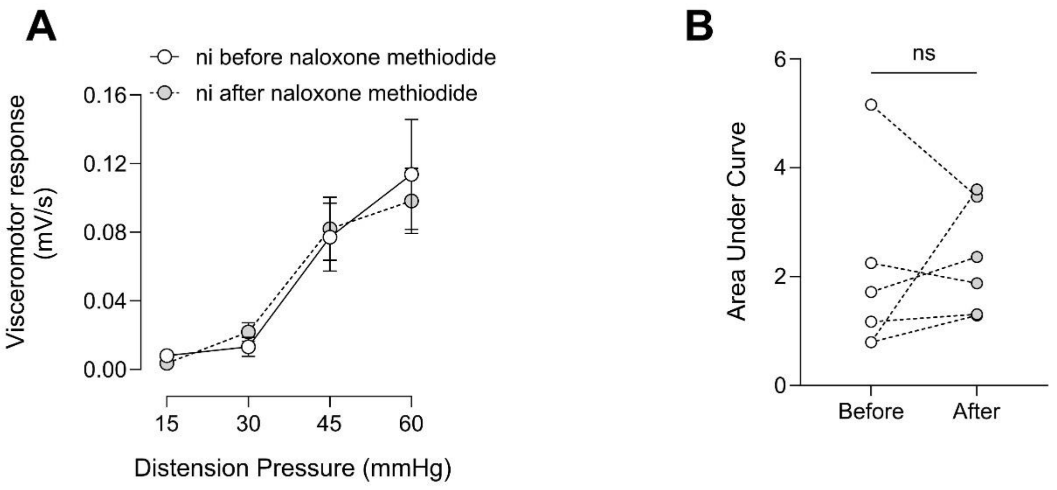


**S4 Fig (related to Fig 4): Naloxone methiodide treatment has no impact on visceral nociceptive responses at steady state**

**(A and B)** Colorectal distension was performed to assess visceral sensitivity of each mouse before (in white) or 30 min after (in gray) intraperitoneal injection of naloxone-methiodide. **(A)** Visceromotor response (VMR) in response to increasing colorectal distension pressure (15 to 60mmHg) was measured in uninfected mice (ni) treated or not with Naloxone methiodide. Data are shown as mean +/- SEM and are from 1 experiment representative with n = 6 mice. **(B)** Area Under the Curve (AUC) are represented for each mouse with 2 dots corresponding to the AUC before (in white) and after (in gray) naloxone methiodide treatment. Statistical analysis was performed using a Wilcoxon's test.
