# Supplementary material for: Toxoplasma gondii chronic infection decreases visceral nociception through peripheral opioid receptor signaling
Source: PLoS Pathog. 2025 Apr 29;21(4):e1013106. doi: 10.1371/journal.ppat.1013106 (PMC12068698; doi:10.1371/journal.ppat.1013106)
Supplement: S5 Fig — (A and B) Combination of flow cytometry analysis and FISH detection of enkephalin encoding mRNA (Penk mRNA) in different colonic cell types in non-infected (white) and chronically infected mice (ip, orange). (A) Gating strategy used to identify the different cell subsets in the colon. Cell doublets were excluded using double gating on SSC-A vs. SSC-H followed by SSC-W vs. SSC-H exclusion. Dead cells were removed using a viability marker to allow analysis of live cells only. Arrows indicate the order of the gating strategy. (B) Flow cytometry analysis of the indicated cell subsets in the colon of non-infected (ni) vs. mice chronically ip-infected for 70 days (T. gondii). Box and whisker show median + /- IQR of the percentage of Penk-expressing cells in the cell subset indicated above each graph. Data are from 1 experiment and each dot represents one mouse with a total of 5 non-infected (ni) and 7 chronically ip-infected (T. gondii) mice. (PDF) [file ppat.1013106.s005.pdf]

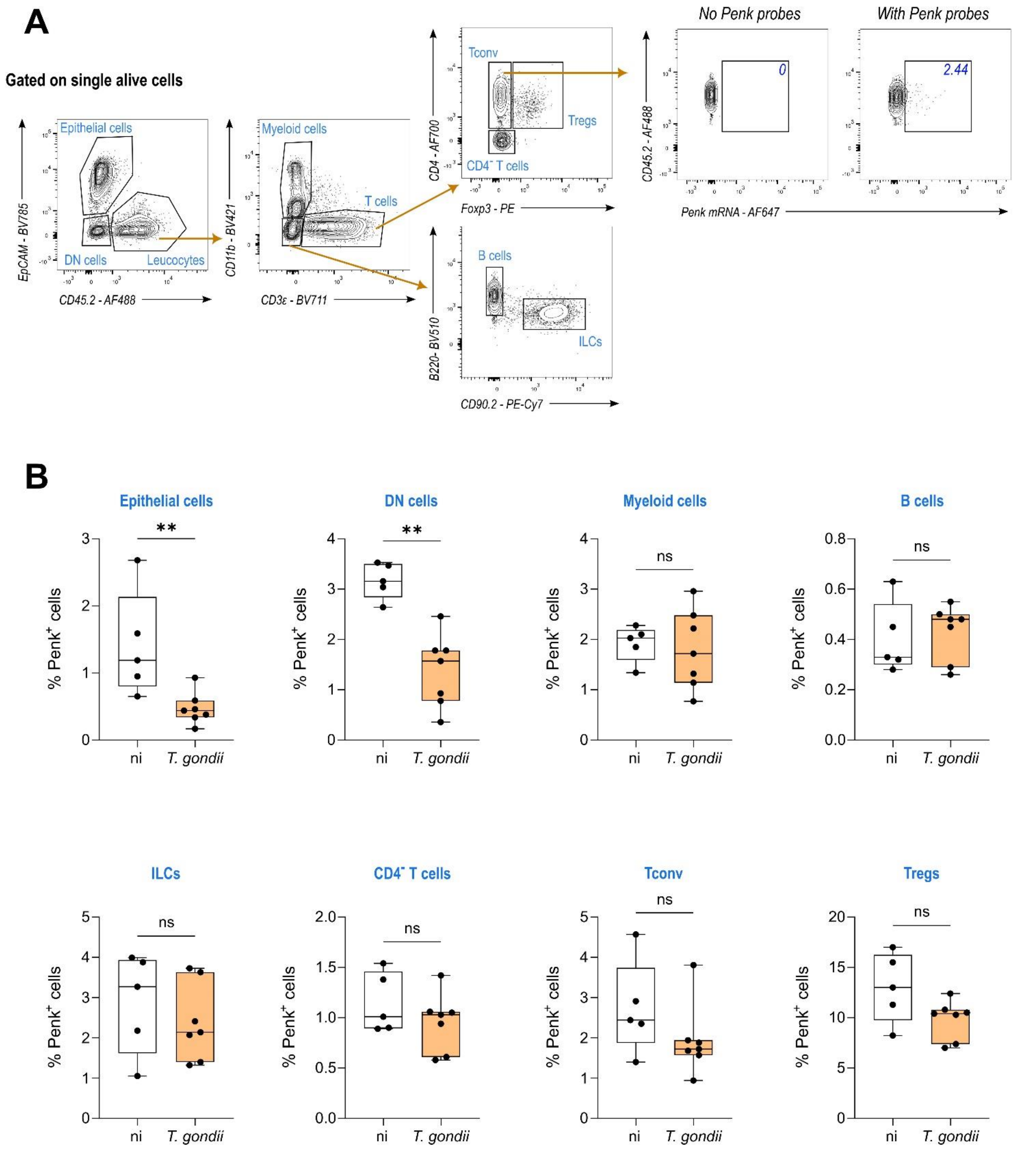

S5 Fig. Chronic infection by *T. gondii* does not increase *Penk* mRNA expression in colonic cell subsets

(**A** and **B)** Combination of flow cytometry analysis and FISH detection of enkephalin encoding mRNA (*Penk* mRNA) in different colonic cell types in non-infected (white) and chronically infected mice (ip, orange). **(A)** Gating strategy used to identify the different cell subsets in the colon. Cell doublets were excluded using double gating on SSC-A vs. SSC-H followed by SSC-W vs. SSC-H exclusion. Dead cells were removed using a viability marker to allow analysis of live cells only. Arrows indicate the order of the gating strategy. **(B)** Flow cytometry analysis of the indicated cell subsets in the colon of non-infected (ni) vs. mice chronically ip-infected for 70 days (*T. gondii*). Box and whisker show median +/- IQR of the percentage of *Penk*-expressing cells in the cell subset indicated above each graph. Data are from 1 experiment and each dot represents one mouse with a total of 5 non-infected (ni) and 7 chronically ip-infected (*T. gondii*) mice.
