# Supplementary material for: Toxoplasma gondii chronic infection decreases visceral nociception through peripheral opioid receptor signaling
Source: PLoS Pathog. 2025 Apr 29;21(4):e1013106. doi: 10.1371/journal.ppat.1013106 (PMC12068698; doi:10.1371/journal.ppat.1013106)
Supplement: S6 Fig — (A-C) Taxonomic analysis of the colon mucosa microbiota of latently infected (T. gondii, 70 dpi) vs. uninfected (ni) mice with (A) Euclidean distance-based Principal Component Analysis (PCA); (B) linear discriminant analysis (LDA) scores showing the 2 microbial taxa that are significantly enriched in uninfected mice; (C) diversity indices. (D-G) Inferred functional analysis of the colon mucosa microbiota of latently infected (T. gondii, 70 dpi) vs. uninfected (ni) mice with (D) Euclidean distance-based PCA; (E) LDA scores showing the 2 microbial pathways significantly enriched in uninfected mice; (F) Kyoto Encyclopaedia of Genes and Genomes (KEGG)-based PCA and (G) Enzyme Commission (EC)-based PCA. In (C), statistical analysis was done with 2-way-ANOVA followed by the 2-step linear procedure of Benjamini, Krieger and Yekutieli to correct for multiple comparisons by checking false discovery rate (<0.05). *P < 0.05, **P < 0.01. In (A, D, F, G), statistical differences in PCA were evaluated with 1-way perMANOVA non-parametric test. Data are from 1 experiment with n = 5 mice per group. (PDF) [file ppat.1013106.s006.pdf]

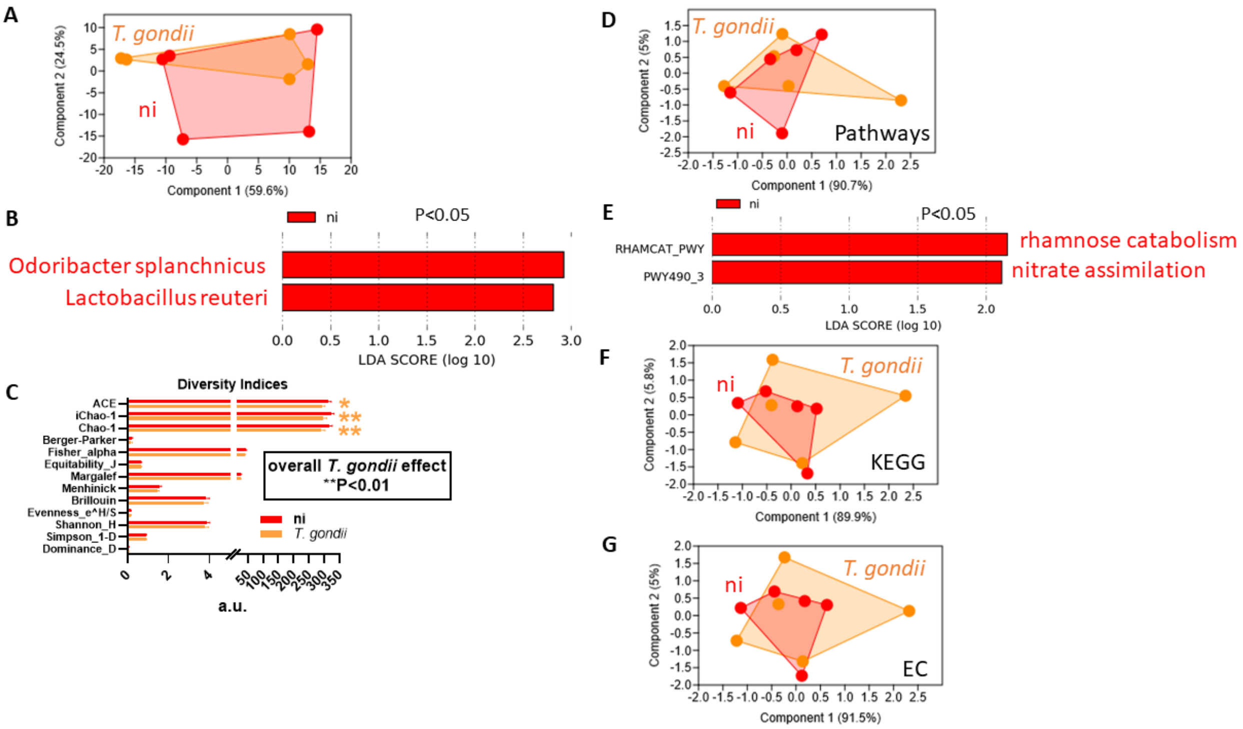

S6 Fig. Changes in the colon mucosa-associated microbiota and inferred microbiome functions, induced by *T. gondii* chronic infection

(A-C) Taxonomic analysis of the colon mucosa microbiota of latently infected (*T. gondii,* 70 dpi) vs. uninfected (ni) mice with **(A)** Euclidean distance-based Principal Component Analysis (PCA) ; **(B)** linear discriminant analysis (LDA) scores showing the 2 microbial taxa that are significantly enriched in uninfected mice ; **(C)** diversity indices. **(D-G)** Inferred functional analysis of the colon mucosa microbiota of latently infected (*T. gondii,* 70 dpi) vs. uninfected (ni) mice with **(D)** Euclidean distance-based PCA ; **(E)** LDA scores showing the 2 microbial pathways significantly enriched in uninfected mice ; **(F)** Kyoto Encyclopaedia of Genes and Genomes (KEGG)-based PCA and **(G)** Enzyme Commission (EC)-based PCA. In **(C),** statistical analysis was done with 2-way-ANOVA followed by the 2-step linear procedure of Benjamini, Krieger and Yekutieli to correct for multiple comparisons by checking false discovery rate (<0.05). *P<0.05, **P<0.01. In **(A, D, F, G)**, statistical differences in PCA were evaluated with 1-way perMANOVA non-parametric test. Data are from 1 experiment with n=5 mice per group.
