# Supplementary material for: Toxoplasma gondii chronic infection decreases visceral nociception through peripheral opioid receptor signaling
Source: PLoS Pathog. 2025 Apr 29;21(4):e1013106. doi: 10.1371/journal.ppat.1013106 (PMC12068698; doi:10.1371/journal.ppat.1013106)
Supplement: Attachment — Submitted filename: Comment.docx [file ppat.1013106.s007.docx]

The authors' work provides the detailed mapping of immune changes in the colon during latent infection and reveals a new consequence of T. gondii chronic infection: a decreased visceral nociceptive response to colorectal distension. Overall, the article is convincing, with solid experiments and high-quality writing. However, the following points need improvement before the manuscript can be considered for publication.

**Major:**

The authors suggest that "Toxoplasma gondii chronic infection decreases visceral nociceptive responses through peripheral opioid signaling." Given that opioids are analgesic molecules, it would be expected that administering antagonists to opioid receptors would exacerbate pain phenotypes in pain models, even in the absence of infection. Consequently, in Figures 4B and 4C, the comparison between only two groups was insufficient to conclusively demonstrate that the observed reduction in visceral pain during infection is mediated by opioid signaling. To strengthen this claim, the authors should consider including additional experimental groups and conducting further experiments.

**Minor:**

During chronic infection, despite elevated enkephalins released by Trm, is there a statistically significant increase in the tissue content of enkephalins? Additionally, does the content of enkephalins in the tissue significantly decrease after the knockout of enkephalins secreted by Trm?

Would it be possible to measure Penk mRNA expression in other immune cells via flow cytometry, similar to what was done in Figure S3B? If other immune cells besides CD4+ T cells have the potential to release enkephalins, this would be valuable information.

Figure 1F does not indicate the number of mice used, each figure legend should clearly state that.
