# Supplementary material for: Toxoplasma gondii chronic infection decreases visceral nociception through peripheral opioid receptor signaling
Source: PLoS Pathog. 2025 Apr 29;21(4):e1013106. doi: 10.1371/journal.ppat.1013106 (PMC12068698; doi:10.1371/journal.ppat.1013106)
Supplement: Attachment — Submitted filename: Audibert_R1_response_to_reviewers_v8.docx [file ppat.1013106.s009.docx]

We first wish to thank the 3 reviewers for their useful comments and insightful suggestions, which helped us to strongly improve the manuscript. Please find below a point-by-point response to all their concerns (copied as blue text).

**Part I - Summary**

Please use this section to discuss strengths/weaknesses of study, novelty/significance, general execution and scholarship.

Reviewer #1: The authors' work provides the detailed mapping of immune changes in the colon during latent infection and reveals a new consequence of T. gondii chronic infection: a decreased visceral nociceptive response to colorectal distension. Overall, the article is convincing, with solid experiments and high-quality writing. However, the following points need improvement before the manuscript can be considered for publication.

Reviewer #2: T. gondii is a food-borne parasite that commonly infects humans. Upon ingestion of oocysts or tissue cysts, sporozoites or bradyzoites are released, which convert into tachyzoites that pass the epithelial barrier. After reaching the lamina Propria, they disseminate throughout the body. Intestinal inflammation, mainly driven by Th1 cytokines, is observed with high doses of tissue cysts upon oral infection in the acute phase of infection in certain inbred mouse strains (BL/6 e.g.) and is primarily localized to the distal part of the small intestine. These lesions revert to normal during the chronic phase of infection and resemble those associated with inflammatory bowel disease (IBD, particularly Crohn´s disease).

In the present study by Audibert et al., the Blanchard lab addressed the question to what extend T. gondii promotes the development of PI-IBS (Irritable Bowel Syndrome, not to be confused with IBD) by influencing the neuroimmune environment of the gut. To this end, the effects of T. gondii on the microenvironment of the colon and on visceral nociceptive responses were investigated in a mouse model of chronic infection.

In general, the quality of the data is good, however, certain experiments should be repeated and/or more animals included. The decrease in visceral nociception in chronically infected mice is interesting but an explanation of the mechanism and cell types involved its missing. Without these data, the paper is rather descriptive and does not advance the field sufficiently.

After initially comparing i.p. and oral infections, the authors subsequently decided to use only tachyzoites for the remainder of the study, as this approach allows for a reduction in the number of donor animals required. This is certainly commendable; however, in my opinion, the decision to use the i.p. model instead of the oral model reduces the physiological relevance of the study. I suggest adding colon thickness and length in Figures 1E and 1F for the oral model of infection. Including this data would help to rule out macroscopic differences between both infection models and would give more weight to the decision to select the i.p. model of infection.

These are my two major concerns with the present study.

We understand the reviewer’s concern regarding the choice to work with tachyzoites injected intra-peritoneally instead of cysts administered *per os*. Beyond the benefit of reducing the number of animals used in this study, there are 3 additional arguments to justify this choice:

1) Similar to what we observed after tachyzoite ip infection, *per os* cyst infection did not lead to macroscopic inflammation of the colon at chronic stage, as depicted in the new **Fig 1E, 1F** (lines 135-141);
2) Following cyst oral ingestion, experimental models suggest that the primary site of host invasion, before disseminating to other tissues, is the small intestine (from the duodenum [*Rahman et al., Front Cell Infect Microbiol. 2020 Apr 16;10:161. doi: 10.3389/fcimb.2020.00161 PMID 32373554*], to the jejunum [*Gregg et al., Infect Immun. 2013 May;81(5):1635-43. doi: 10.1128/IAI.01126-12 PMID 23460516*] and the ileum [*Coombes et al., Proc Natl Acad Sci U S A. 2013 May 21;110(21):E1913-22. doi: 10.1073/pnas.1220272110 PMID 23650399]*), but not the colon. Therefore, we hypothesize that effects detectable at chronic stage in the colon are more likely to result from secondary events following systemic dissemination than to originate from primary lesions/changes related to initial host invasion. In that sense, the tachyzoite ip model appears adequate to reproduce this setting. We have added these considerations in the discussion (lines 327-331).
3) We observed that the injection of naloxone methiodide also increased visceral sensory responses in *per os*-infected mice (see **Fig 4C** in the revised version of the manuscript, lines 280-284), demonstrating the implication of peripheral opioid receptor signaling in this setting as well, thereby strongly suggesting that the same analgesic mechanism operates after oral and ip infection.

Moreover, we would like to reiterate that our study is innovative for the field and not purely descriptive. Although we do not formally establish the complete mechanism, we provide critical mechanistic elements to explain the hyposensitive phenotype induced by *T. gondii* chronic infection. We show 1) that this effect is driven by signaling through peripheral (and not central) opioid receptors, thanks to the use of naloxone methiodide (see **Fig. 4B, 4C**), and 2) that enkephalins produced by T cells are not involved in this process, thanks to a novel T cell-specific conditional KO of the *Penk* gene that was developed for this study (see **Sup. Fig. S3**). Lastly, additional experiments included in the revised version of the manuscript also reveal that the hyposensitive phenotype is not accompanied by drastic changes in the colon mucosa microbiota, suggesting that this phenotype is independent of bacterial community shifts (**Sup. Fig. S6**), lines 301-319.

Reviewer #3: The manuscript by Audibert et al. sets out to determine if Toxoplasma infection could be a pathogen that triggers PI-IBS. Several gastrointestinal pathogens have been shown to play a role in instigating PI-IBS and because Toxo has been shown to cause substantial inflammation that is a kin to Crohn’s disease the authors set out to determine if it could also participate in IBS. However, these are two very different diseases with IBD being driven by inflammation directed at the microbiota and IBS having alterations in the function and pain response of the gut. The study uses the PruGRA6OVA model of Toxo which results in latency in B6 mice. The authors characterize the chronic immune environment in the colon of the mice infected by both IP and PO and find that little to no parasite exists during the chronic phase of infection. They also test the VSM to determine the nociceptor response in the colon and surprisingly find it reduced. They go on to determine if the Penk, a precursor to enkephalin, is involved in the dampening of the nociceptor pain response. They use a T cell specific Penk system to test if it is derived from T cells but it is not. It was difficult to understand the justification for the T cells specific KO of Penk.

We apologize if the justification was difficult to understand. The justification that was initially provided was the following: “studies have highlighted that effector T cells, in particular CD4+ T cells, can release endogenous opioid peptides called enkephalins, that are able to alleviate pain in different inflammatory contexts, including colitis. Furthermore, it was reported that enkephalins could be produced by colitogenic T cells, highlighting the dual effect of T cells on inflammation and pain modulation *[Boue et al. Gastroenterology. 2014 Jan;146(1):166-75. PMID 24055279 ; Basso et al. 2016 J Neuroinflammation. 2016 Jun 1;13(1):132 PMID 27245576]*. Based on this, we hypothesized that the release of enkephalins by T cells present in the colon of chronically infected mice may be responsible for the decrease in visceral nociceptive responses”.

To increase clarity, we have added the following information in the revised version: “Notably, transcripts of Penk, the gene coding for enkephalins, were the only detectable mRNA of endogenous opioids in the colon tissue (**Sup. Fig. S3A**)” (see lines 241-243).

They also block all mu opioid receptor and show that rescues the pain response only in the infected animals. This study is of interest in understanding how chronic infection and inflammation can alter a tissue and its function long-term. There are strengths to the study, however it seems premature in some of the major conclusions, ie Toxo could be negatively associated with IBS and there are some experiments that could be done to deepen the understanding of how this system is being altered that the authors have proposed in the discussion but have not done yet.

We agree that it would be premature to conclude that *T. gondii* infection is negatively associated with IBS, this is why we did not draw a definitive conclusion in our manuscript but rather speculated about the relationship with abdominal pain (the main clinical sign of IBS, though not the only one, which was more specifically assessed in our work). What we had written at the end of the abstract was: “*They suggest that in contrast to other gut microbes, T. gondii infection could be negatively associated with abdominal pain*” and at the end of the discussion: “*These data support the notion that T. gondii is likely not a driver of post-infectious IBS, and they even suggest that latent T. gondii infection could be negatively associated with abdominal pain in humans.*”

We have now reformulated the last sentence of the discussion as such: “Our study suggests that *T. gondii* is unlikely to be a major contributor to post-infectious IBS and may instead indicate a potential negative association between latent *T. gondii* infection and abdominal pain.” (see lines 418-420 of the discussion).

The study as it stands is of interest in how this infection impacts the pain response, however there is limited mechanistic data, and the data supporting Toxo and IBS connection seems premature, together this limits the enthusiasm for the overall findings as it stands.

The relationship between *T. gondii* infection and IBS is addressed above.

Moreover, we would like to specify that although naloxone has the highest affinity for mu-opioid receptors (MOR), it is able to block all 3 classical opioid receptors (mu, delta and kappa). Therefore, using naloxone methiodide, we block the overall opioid signaling in the peripheral compartment.

We acknowledge that we did not decipher the complete mechanism behind the hypoalgesic phenotype induced by *T. gondii* chronic infection. Nevertheless, we uncovered critical mechanistic elements, as stated above, making our findings both innovative and remarkable, in particular since gastrointestinal pathogens are typically thought to exacerbate rather than mitigate visceral sensitivity.

Part II – Major Issues: Key Experiments Required for Acceptance

Please use this section to detail the key new experiments or modifications of existing experiments that should be absolutely required to validate study conclusions.

Generally, there should be no more than 3 such required experiments or major modifications for a "Major Revision" recommendation. If more than 3 experiments are necessary to validate the study conclusions, then you are encouraged to recommend "Reject".

Reviewer #1: The authors suggest that "Toxoplasma gondii chronic infection decreases visceral nociceptive responses through peripheral opioid signaling." Given that opioids are analgesic molecules, it would be expected that administering antagonists to opioid receptors would exacerbate pain phenotypes in pain models, even in the absence of infection. Consequently, in Figures 4B and 4C, the comparison between only two groups was insufficient to conclusively demonstrate that the observed reduction in visceral pain during infection is mediated by opioid signaling. To strengthen this claim, the authors should consider including additional experimental groups and conducting further experiments.

We understand that reviewer #1 would like to see the effects of peripheral opioid receptor blockade on the visceromotor response (VMR) of uninfected mice upon colorectal distension. These results were already included as **Sup Fig. S4** of the initial manuscript. These results show that naloxone methiodide treatment has no impact on VMR at steady-state in non-infected mice, a finding that has been previously reported in the literature (see Fig. 4A of *Basso et al., Pain. 2018 Feb;159(2):331-341. doi: 10.1097/j.pain.0000000000001103. PMID 29140925* ; Fig. 4D of *Basso et al., Neurogastroenterol Motil. 2020 Feb;32(2):e13743. doi: 10.1111/nmo.13743. PMID: 31588671* ; Fig. 5C of *Boué et al., Gastroenterology. 2014 Jan;146(1):166-75. doi: 10.1053/j.gastro.2013.09.020. PMID 24055279*). Thus, **Sup. Fig. S4** confirms these results in the settings of our study and indicate that opioidergic signaling in the colon is not active in physiological conditions. In contrast, we hypothesize that chronic infection by *T. gondii* induces the release of endogenous opioids in the colon that lead to the decreased visceral nociception that we observed in our study. We have added the above references in the manuscript to improve clarity of the corresponding paragraph (lines 283-284).

To summarize, in the current study, we measured the biological effect of naloxone methiodide on 2 experimental groups: latently infected mice (**Fig. 4B, 4C**) and uninfected mice (**Sup. Fig. S4**), with the latter confirming the absence of effect at steady-state in uninfected mice. Since the data in uninfected mice are rather “confirmatory”, we still do not think that it would be ethically justified to perform additional experiments with more uninfected animals.

We are confident that altogether, the provided data are compelling enough to state that the hypoalgesic phenotype developed during latent *T. gondii* infection is caused by peripheral opioid receptor signaling.

Reviewer #2: Fig. 1B

The authors report how many animals were used in this study, but did all the animals survive the infection? If not, are there significant differences between the respective routes of infection? And were all the animals - especially those showing no weight loss - really infected? A conventional test for seroconversion would be necessary to confirm this.

As usual with *T. gondii* experimental infection of laboratory mice, some animals did not survive acute infection, with a survival rate that was slightly higher after *per os* (~ 93%) than *ip* infection (~ 80%). Importantly, in the course of this study, we assessed brain parasite load by qPCR on genomic DNA in every infected animal at euthanasia, and we excluded any mouse that had no detectable parasite load in the brain. By confirming the presence of *T. gondii* in the brain with such a direct assay, we ensured that all data presented in this manuscript originate from genuinely infected mice. We have added this precision in the Material and Methods section, see lines 435-436.

Based on the reviewer’s comment, we assume that there may be a misconception. **Fig. 1B** depicts the aggregation of different cohorts of mice used in this study, highlighting weight loss over the course of infection, a parameter that was systematically measured in all mice post-infection. However, we did not systematically perform all readouts in all mice (for example, in some experiments we performed only colorectal distension and histology, while other experiments were dedicated to flow cytometry), which explains why the other graphs do not display as many dots as **Fig. 1B**.

Fig. 1C

Were the animals that were infected in 1B examined here? Does this mean that, in the case of the oral infection, 10 animals died spontaneously?

As stated above, parasite load in the brain was systematically assessed at euthanasia, but not parasite load in the colon. For consistency, **Fig. 1C** only shows the results from experiments in which parasite load was assessed in both the brain and the colon, explaining why the number of mice is reduced compared to **Fig. 1B**.

Fig. 1D

Here, data from three independent repetitions are pooled, with 15, 6, and 12 animals respectively. Are these the respective animal numbers per repetition, or the total numbers? If the former, please specify. If the latter, then significantly more animals need to be included per repetition (especially for the oral infections), also in order to make statistically robust conclusions.

These numbers are indeed the total numbers of mice per group, as now specified in the legend of **Fig. 1D** (line 155). We did perform an additional experiment in the *per os* setting (see **Fig. 1 for the reviewer** below), which revealed a similar hyposensitive response to colorectal distension as in ip-infected mice, reinforcing the robustness of our phenotype that develops whatever the route of *T. gondii* infection.

**Figure 1 for the reviewer:**

**(A)** Visceromotor response (VMR) to increasing colorectal distension pressures (15 to 60 mm Hg) measured in non-infected (ni) mice or *T. gondii*-infected mice infected either *per os* or ip. Data are shown as mean +/- SEM and correspond to the figure 1D of the manuscript. Statistical analysis was performed using a mixed-effects model with Sidak’s multiple comparison test. * indicates a statistically significant difference between ni and po groups, # indicates a statistically significant difference between ni and ip groups. **(B)** VMR to increasing colorectal distension pressures (15 to 60 mm Hg) measured in non-infected (ni) mice vs. mice infected *per os*. Data are shown as mean +/- SEM and correspond to 1 experiment with n = 7 (ni) and n = 11 (po) mice. Statistical analysis was performed using a mixed-effects model with Sidak’s multiple comparison test.

Fig. 1E

Why are the pooled data from both (two) experiments not shown in this case?

As mentioned above, determining colon thickness and length following oral infection is necessary in order to (better) justify the subsequent approach (i.p. infection).

Thank you for your vigilance, there was a mistake in the legend. Data were indeed pooled from both experiments. As requested, we have now added the measurements of colon length and thickness at chronic stage in the oral infection model as well (see new **Fig. 1E, 1F**), showing that, similar to what we observed after tachyzoite ip infection, *per os* infection with cysts did not lead either to macroscopic inflammation of the colon at chronic stage (see new **Fig. 1E**, integrating pooled data from 2 independent experiments, lines 135-141). In addition, HE-stained colonic tissue from *per os* *T. gondii*-infected mice displayed no alteration compared to non-infected mice, illustrating the absence of microscopic inflammation after oral infection (new **Fig. 1F**).

Fig. 4B

Again here, why are the pooled data from both (two) experiments not shown in this case?

We apologize for the inaccuracy of the figure legend. We did perform the experiments with naloxone methiodide twice but the design of both experiments was not strictly identical, therefore it would not be fair to pool both experiments in a single figure. In one case (previously depicted on **Fig. 4B**), VMR was measured before-after naloxone methiodide injection in the same animals, while in the other case (not shown in the first version), latently infected mice were divided in 2 groups, with one that was injected with naloxone methiodide and the other with vehicle, before VMR assessment. In this second experiment, mice had been infected orally, which indicates that peripheral opioid signaling is involved in the hyposensitive phenotype independently of the mode of infection, thereby strengthening the robustness of our results. To consolidate our study, **Fig. 4** (panels **B & C**, see lines 280-284) now shows the 2 experimental settings resulting in a similar outcome, which is that naloxone methiodide reverses the decrease in VMR to colorectal distension during latent *T. gondii* infection.

Fig. S3

There seems to be an issue with the labeling of the individual panels here. Please correct accordingly.

This experiment should be repeated in order to ultimately present the pooled data (from at least two experiments).

Thank you for your vigilance. The panel letters and figure legend have been corrected.

Data of **Sup Fig. S3** validate the T cell-specific KO of Penk gene by FlowFISH (**Sup. Fig. S3B, S3C**), and show that *T. gondii*-induced decrease in nociceptive responses is independent of T-cell-derived enkephalins (**Sup. Fig. S3D-F**). This negative result is actually corroborated by new FlowFISH data showing that *Penk* gene expression is not increased (it is in fact rather decreased) in T cells during latent *T. gondii* infection (see new **Sup. Fig. S5** and lines 272-274).

As the results of **Sup. Fig. S3** ruling out a major role of T cell-produced enkephalins in the phenotype are somewhat “negative” and are in line with the absence of increase of *Penk* gene expression in T cells as assessed by FlowFISH (see new **Sup. Fig. S5**), and since these data are not presented as a main figure, we do not think it would be justified to repeat such experiments.

Fig. S4

As an important control for Fig. 4, data from more than just a single experiment with 6 mice should be included here.

We agree that this is an important control. Yet, as stated above, the results of **Sup. Fig. S4**, i.e. the absence of effect of naloxone methiodide at steady-state, was expected and confirmatory of previously published observations (see Fig. 4A of *Basso et al., Pain. 2018 Feb;159(2):331-341. doi: 10.1097/j.pain.0000000000001103. PMID 29140925* ; Fig. 4D of *Basso et al., Neurogastroenterol Motil. 2020 Feb;32(2):e13743. doi: 10.1111/nmo.13743. PMID: 31588671* ; Fig. 5C of *Boué et al., Gastroenterology. 2014 Jan;146(1):166-75. doi: 10.1053/j.gastro.2013.09.020. PMID 24055279)*. Hence, we doubt that it would be scientifically and ethically justified to perform additional experiments with more animals in order to further replicate this finding.

Reviewer #3: 1. The authors do not show the data that Penk is the only one detected or what tissues it is detected in, what is the level in the colon? In various cells from the colon? What about the brain? This data is stated in the discussion but not shown and is critical for understanding the expression and how infection alters this expression. The authors have the FlowFish worked out and showed it nicely in the KO mice.

We fully agree that such data are critical to start deciphering the mechanism.

We added as new **Sup Fig. S3A**, qPCR data quantifying the mRNA expression of *Penk* gene coding for enkephalins, *Pdyn* gene coding for dynorphin and *Pomc* gene coding for proopiomelanocortin/β–endorphin in the colon. These data provide evidence that *Penk* transcripts are the only detectable mRNA for endogenous opioids in the infected colonic tissue (see lines 241-243), further justifying the choice to focus on enkephalins in the rest of the study. We did not investigate opioid gene expression in the brain since, based on the effect of naloxone methiodide which cannot cross the blood-brain barrier, hypoalgesia is more likely caused by local opioid receptor signaling in the colon than in the central nervous system.

We also added new FlowFISH data to determine the level of *Penk* transcripts in various cells isolated from the colon (see new **Sup. Fig. S5**). These new data show that latent *T. gondii* infection does not lead to an increase in *Penk* gene expression in any of the cell subset analyzed. In contrast, we observed a global trend for a reduction of *Penk* gene expression in the infected colon.

As FlowFISH only estimates the amount of mRNA, these results are not necessarily contradictory with the conclusion drawn from the naloxone methiodide experiments that peripheral opioid receptor signaling drives the hypoalgesic phenotype. As discussed (lines 367-379), it would be essential to quantify changes in bioactive opioid peptides in the colon microenvironment upon chronic *T. gondii* infection.

With the aim to analyze Penk at the protein level, we purchased two different anti-PENK antibodies targeting the Penk precursor (pro-Enkephalin) or the bioactive peptides (PA1-30928, ThermoFisher and sc67044, Santa Cruz). We tested them in immunofluorescence and flow cytometry approaches, using WT *vs*. genetically Penk-deficient full KO mice (B6.129-Penk-rstm1Pig/J strain, Jackson Laboratory, see *König et al., Nature. 1996 Oct 10;383(6600):535-8. doi: 10.1038/383535a0* ; and *Mas-Orea et al., Commun Biol. 2023 Nov 16;6(1):1168. doi: 10.1038/s42003-023-05542-2*). We performed whole-mount IF stainings of colonic myenteric plexus, where some neurons are known to express *Penk*. After mechanical removal of the colonic mucosal layer, confocal imaging revealed a positive signal that was detected in myenteric neurons of both WT and Penk KO mice (**Fig. 2A for the Reviewer** below, panel A). Moreover, using flow cytometry, we also obtained aspecific profiles on *in vitro*-activated CD4 T cells, which are known to produce high amount of enkephalin (**Fig. 2B for the Reviewer** below). At last, it was impossible to differentiate low enkephalin-producing cells such as CD8 T cells, from high producing cells like CD4+ Tregs (see **Fig. 2C for the Reviewer below**).

**Figure 2 for the reviewer:**

**(A)** Assessment of the specificity of 2 antibodies targeting the precursor of enkephelin (pro-enkephalin, Penk) by immunofluorescence. Colons from WT or Penk KO mice were harvested, washed in HBSS and opened longitudinally. The mucosal layer was removed by mechanical dissection under a binocular magnifier. Following fixation in PFA 4%, the mucosa-free tissues were submitted to whole-mount immunofluorescence stainings using 2 different anti-Penk antibodies (purple) combined with the neuronal marker anti-substance P (green). Nuclei were stained with DAPI. Tissues were mounted between slides and coverslips in Vectashield mounting medium and observed with Zeiss LSM 710 confocal microscope. **(B and C)** Assessment of the specificity of the anti-Penk antibody sc67044 (Santa Cruz) by flow cytometry in *in vitro*-activated splenic CD4 T cells **(B)** or in different T cell subsets from the colon **(C)** isolated from WT *vs*. Penk KO mice.

Our data clearly indicate that none of the tested antibodies provided specific Penk labeling, making them unsuitable for further use in antibody-based approaches and thus preventing us from localizing the Penk-producing cells in the colon or quantifying bioactive Penk peptides.

The most compelling data is the opioid receptor inhibition but without supporting data to understand the source of Penk it is difficult to understand what is the source driving the Penk increase.

As mentioned above and now added in the discussion (lines 367-379), several hypotheses can be envisaged to reconcile the apparent paradox between an opioid receptor-mediated hypoalgesia and a reduction in Penk mRNA in colon-isolated cells:

(i) various cell types are able to produce opioids, including neurons, which may not be as amenable for isolation and flow cytometry analysis as lymphocytes. A heightened opioid secretion by such cells, or by another cellular population that is not easily visualized by flow cytometry and was not evaluated here, may underlie the increased opioid receptor activity ;

(ii) independently from mRNA expression level, hypoalgesia may ultimately arise from post-translational changes in opioid peptide precursors leading to increased processing and/or increased secretion of the final bioactive peptides. Quantifying changes in bioactive opioid peptides in the colon microenvironment upon chronic *T. gondii* infection would be useful but the absence of reliable tools to perform this at the tissue and single-cell levels was a major roadblock preventing us from tackling this question ;

(iii) at last, a closer proximity between opioid-secreting cells and nociceptive fibers of the colon may also contribute to reinforce the biological effects of these labile molecules. Here again, due to the lack of reliable tools, protein-based approaches such as immunolocalization studies are not currently possible.

2.In the IP route what do the authors know of the contribution of the adrenal gland to the production of Penk/Enkephalins? This is also a major source in the body but is not considered in the IP infection model, again does Penk increase in the adrenal gland over the course of infection?

It is true that some papers have reported that cells from the adrenal gland can produce proenkephalin and enkephalins. We did not consider this phenomenon in our study since 1) free bioactive enkephalin peptides are known to be rapidly degraded in the bloodstream and in tissues (see *Dupont et al., Life Sci. 1977 Oct 1;21(7):907-14. doi: 10.1016/0024-3205(77)90254-5 PMID 927020 ; Vandenberg et al., Arch Biochem Biophys. 1985 Nov 1;242(2):515-22. doi: 10.1016/0003-9861(85)90238-3 PMID 4062293*) and 2) our previous work indicated that the activation of opioid receptors on sensory neurons controlling visceral sensitivity depends on focal and local secretion of opioid peptides (see *Mas Orea et al., J Neuroinflammation. 2022 Jan 6;19(1):7. doi: 10.1186/s12974-021-02352-3. PMID: 34991641 ; Mas Orea et al., Commun Biol. 2023 Nov 16;6(1):1168. doi: 10.1038/s42003-023-05542-2. PMID: 37968381 ;* Fig. S1 of *Basso et al., J Gastroenterol. 2018 Feb;53(2):215-226. doi: 10.1007/s00535-017-1341-2. PMID: 28424989 ; Basso et al., J Neuroinflammation. 2016 Jun 1;13(1):132. doi: 10.1186/s12974-016-0591-x. PMID: 27245576*).

3 What is the percentage of the TRM in CD4 and CD8 that are specific to OVA and or Toxo? It is hard to interpret the conclusions of the gut as it is not clear the parasite specific TRM compared to the commensal/other TRM. Also, it is difficult to make broad conclusions from other studies on these due to the influence of vendor and animal facilities.

From a basic point of view, we agree that it would be interesting to quantify the respective proportion of commensal-specific vs *T. gondii* (OVA)-specific T cells or Trm. Using *in vitro* restimulation with *T. gondii*-infected dendritic cells, we could document the presence of resident parasite-specific CD4+ T cells in the colon of latently infected mice and not uninfected mice (see **Fig. 3 for the reviewer**). Unfortunately, this restimulation assay was not as clear for CD8+ T cells and our labeling with OVA peptide-loaded H-2K^b^ dextramer did not work as well as in the brain. In addition, we unfortunately could not detect commensal-specific T cells by restimulation with commensal bacteria-loaded dendritic cells. Therefore, at this stage, we are unable to provide compelling data on the respective proportions of colonic T cells specific for the parasite (OVA) vs. the microbiota. In any case, we think that this question extends beyond the scope of our study and that our main conclusions hold regardless of the outcome.

We would appreciate if the reviewer could specify which conclusion is hard to interpret and we would be happy to reformulate it.

**Figure 3 for the reviewer:**

**(A to C)** Flow cytometry analysis of cytokine production and resident-memory phenotype of CD4+ FoxP3-negative conventional T cells isolated from the colon of non-infected mice (ni) or chronically ip-infected mice (*T. gondii*) after stimulation by MutuDC (see Fuertes-Marraco et al., Front Immunol. 2012 Nov 7;3:331 PMID 23162549) infected with *T. gondii*. Data are representative of 2 independent experiments and correspond to a pool of T cells sorted from 5 colons for each condition. **(A)** Representative dot plots of IFN-γ and IL-17A production by conventional (Foxp3^-^) CD4^+^ T cells after coculture with MutuDC infected with *T. gondii*. **(B and C)** Percentage of cells producing IFN-γ **(B)** or IL-17A **(C)** in each conventional CD4^+^ T cell subset: resident CD49a+ (right graph) vs non-resident CD49a- (left graph).

4. The discussion could use some more details (line 320-329ish) in the cells that express Penk and cells that express the receptors, these are referred to but it is not the easiest to follow.

As requested, we have now reformulated this paragraph. We hope it will be easier to read in the revised version of the manuscript (lines 367-383).

5. The authors discuss the role of the microbiota in producing signals that can trigger neurotransmitters but do not investigate the dysbiosis in their model or if any of the bugs are in their model. Does antibiotic treatment alter the VSM response in infected mice? This is a straightforward experiment that could answer this question and provide more context into what the authors are studying.

As mentioned in the introduction (lines 96-98) and discussion (lines 399-403), although microbiota dysbiosis is well established in the acute phase of infection, the current literature reports no or only mild dysbiosis during chronic stage of *T. gondii* infection.

Yet, as requested by the reviewer, we investigated the presence of a potential dysbiosis in our model by analyzing the mucosa-associated microbiota in the colon of naïve versus infected mice. As depicted in the new **Sup. Fig. S6** added in the revised version of the manuscript, we found modest changes in the colon mucosa microbiota composition and its predicted function in latently infected compared to uninfected mice, arguing against a major implication of dysbiosis in the observed hypoalgesic phenotype. These new results have been incorporated into the revised manuscript, and we have modified the text accordingly in the Results section (lines 301-319), the discussion (lines 403-412) and the Materials and Methods section (lines 529-544).

As for the use of antibiotics, antibiotic treatment has been shown to alter the visceromotor response to colorectal distension in healthy mice. Indeed, a 10-day course of antibiotics was associated with the emergence of visceral hypersensitivity to CRD (see *Verdu et al., Gut. 2006 Feb;55(2):182-90. doi: 10.1136/gut.2005.066100 PMID 16105890*). For this reason and because we found no major dysbiosis in the chronically infected animals, we respectfully estimated that it was not justified to conduct antibiotic experiments in our study.

6. Is serotonin altered in the gut during chronic infection? As this could also be participating and many stimuli are altered by Toxo infection it seems like this should be measured.

To address this question, we measured the expression level of the genes coding for Tph1 (Tryptophan hydroxylase 1), a rate-limiting enzyme in the biosynthesis of serotonin, and SERT (Serotonin transporter), a transporter responsible for the reuptake of serotonin involved in the negative regulation of serotoninergic signaling. As shown in the **Fig. 4 for the reviewer** below, chronic infection by *T. gondii* led to a decreased expression of *Tph1* and an increased expression of *Sert* in the colon, in comparison to non-infected mice.

**Figure 4 for the reviewer:**

**(A)** Quantification by qPCR of the expression of serotonin metabolism-related genes (*Sert* and *Tph1*) in the colon of non-infected mice (ni) and chronically ip-infected mice (*T. gondii*). Box and whisker show median +/- IQR and data correspond to a pool of 2 independent experiments with each dot representing one mouse. Statistical analysis was performed using an unpaired t test.

These results suggest a decrease in serotonin production/bioavailability in the colon upon latent infection, which would be consistent with an increased activity of the opioid receptor signaling pathway in infected mice, and thus would be in line with the hypoalgesic phenotype associated with infection. At this stage, we feel that these data are too premature and suggestive to be added but we could do so if the reviewers/editor think they would strengthen our manuscript.

Part III – Minor Issues: Editorial and Data Presentation Modifications

Please use this section for editorial suggestions as well as relatively minor modifications of existing data that would enhance clarity.

Reviewer #1: During chronic infection, despite elevated enkephalins released by Trm, is there a statistically significant increase in the tissue content of enkephalins? Additionally, does the content of enkephalins in the tissue significantly decrease after the knockout of enkephalins secreted by Trm?

Importantly, we did not show that Trm release more enkephalins upon latent infection. In fact, as stated above, and now added in the manuscript as new **Sup. Fig. S3A**, we observed that *Penk* transcripts are the only detectable mRNA for endogenous opioids in the colon tissue. Moreover, as measured by FlowFISH (new **Sup. Fig. S5**), *Penk* mRNA levels were unchanged or decreased after infection, arguing against an implication of this opioid in the colon-isolated cell types analyzed by flow cytometry. However, as clarified in the discussion (lines 367-379), the involvement of opioid signaling may occur even in the absence of opioid increase at the mRNA level. We also wish we could quantify the content in enkephalin bioactive peptides in the colon but as explained above, we were prevented from doing so by the absence of reliable tools.

Would it be possible to measure Penk mRNA expression in other immune cells via flow cytometry, similar to what was done in Figure S3B? If other immune cells besides CD4+ T cells have the potential to release enkephalins, this would be valuable information.

Thank you for this insightful suggestion that we followed, see above response to the previous concern, and the new **Sup. Fig. S5**.

Figure 1F does not indicate the number of mice used, each figure legend should clearly state that.

The legend has now been completed.

Reviewer #2: Fig. 2A

In all panels except the first, 12 points for infected animals are displayed, even though the legend states that only 11 animals were used. Please correct the Fig. accordingly.

We appreciate your vigilance. We actually made an error in the submitted figure version.

Initially, we had 12 mice per group (12 uninfected and 12 ip-infected) but due to a technical glitch, we were unable to accurately measure the absolute number of cells in the colon of one infected mouse. However, we forgot to remove this mouse in the graph containing the percentage of cells in the colon, explaining why we had 12 infected mice in the graph showing percentages but only 11 mice in the graph showing absolute numbers.

This mistake was present in both figures 2 and 3, it has been corrected.

Fig. 2C and D

The selection of FACS plots does not seem to effectively represent the data shown. Are there no plots that better exemplify the data presented?

We would be happy to provide better representative Facs plots but we are not sure what is suboptimal here. Could the reviewer provide more details about his/her concern?

Fig. 3A and B

As in Fig. 2A, 12 T. gondii infected animals are shown here, although the legend states 11. Please correct this.

Thank you, the figure has been corrected. The final figure contains 11 dots, for the same reason as **Fig. 2A** (see explanation above).

Reviewer #3: (No Response)

PLOS authors have the option to publish the peer review history of their article (what does this mean?). If published, this will include your full peer review and any attached files.

If you choose “no”, your identity will remain anonymous but your review may still be made public.

Do you want your identity to be public for this peer review? For information about this choice, including consent withdrawal, please see our Privacy Policy.

Reviewer #1: Yes: Yan Li

Reviewer #2: No

Reviewer #3: No

[NOTE: If reviewer comments were submitted as an attachment file, they will be attached to this email and accessible via the submission site. Please log into your account, locate the manuscript record, and check for the action link "View Attachments". If this link does not appear, there are no attachment files.]

Figure resubmission:

While revising your submission, please upload your figure files to the Preflight Analysis and Conversion Engine (PACE) digital diagnostic tool, https://pacev2.apexcovantage.com/. PACE helps ensure that figures meet PLOS requirements. To use PACE, you must first register as a user. Registration is free. Then, login and navigate to the UPLOAD tab, where you will find detailed instructions on how to use the tool. If you encounter any issues or have any questions when using PACE, please email PLOS at figures@plos.org. Please note that Supporting Information files do not need this step. If there are other versions of figure files still present in your submission file inventory at resubmission, please replace them with the PACE-processed versions.

Reproducibility:

To enhance the reproducibility of your results, we recommend that authors of applicable studies deposit laboratory protocols in protocols.io, where a protocol can be assigned its own identifier (DOI) such that it can be cited independently in the future. Additionally, PLOS ONE offers an option to publish peer-reviewed clinical study protocols. Read more information on sharing protocols at https://plos.org/protocols?utm_medium=editorial-email&utm_source=authorletters&utm_campaign=protocols
